# Anaphylaxis to clindamycin following cutaneous exposure

**DOI:** 10.1186/s13223-020-00452-y

**Published:** 2020-06-19

**Authors:** N. Paradis, L. Marois, L. Paradis, F. Graham, P. Bégin, A. Des Roches

**Affiliations:** 1 Pediatric Allergy and Clinical Immunology, CHU Sainte-Justine, 3175 chemin de la Côte-Sainte-Catherine, Montreal, QC H3T 1C5 Canada; 2 Allergy and Clinical Immunology, Centre Hospitalier de l’Universite de Montreal (CHUM), Montreal, Canada; 3 Applied Clinical Research Unit, CHU Sainte-Justine, Montreal, Canada

**Keywords:** Antibiotic anaphylaxis, Atopic dermatitis, Transcutaneous exposition

## Abstract

**Background:**

The role and importance of skin barrier as an immunologic organ and as a potent way of sensitization is well known. However, antibiotics anaphylaxis following skin sensitization has not been reported.

**Case presentation:**

We describe the first case of intravenous clindamycin anaphylaxis, with likely sensitization due to previous topical exposure to clindamycin gel for acne in a 14-year-old boy with history of atopy and mild atopic dermatitis.

**Conclusion:**

This case highlights the potential sensitization to drug allergens, including antibiotics, via the skin.

## Background

Topical application of drugs on disrupted skin is a medical approach widely used in dermatologic conditions [1]. The role and importance of skin barrier as an immunologic organ and as a potent way of sensitization is well described for other allergens like latex, chlorhexidine and also for IgE-mediated food allergy in the context of atopic dermatitis (AD) [2–6]. However, this is the first report of clindamycin sensitization after topical skin treatment that led to anaphylactic reaction upon systemic reexposure.

## Case presentation

A 14 year old boy was admitted to the intensive care unit with suspected staphylococcus toxic shock syndrome. He presented with multiple skin abscesses, fever, headache, macular rash and low blood pressure at 102/49. He was treated with intravenous cefazolin and clindamycin. Five minutes after starting his clindamycin infusion, he complained of throat tightening and dyspnea. Physical examination revealed angioedema, conjunctival hyperemia, generalized hives and wheezing. Saturation decreased to 88%. He was immediately treated with epinephrine, diphenhydramine, salbutamol, hydrocortisone, and symptoms were rapidly controlled except for remaining low diastolic blood pressure. Clindamycin was suspected and discontinued. However, he continued to be febrile and the addition of clindamycin to his penicillin treatment was suggested to inhibit the production of exotoxin associated with toxic shock syndrome, as currently recommended. Considering that infectious episodes can simulate drug allergic reactions, that clindamycin allergy is rare, and that he had never received clindamycin in the past to explain sensitization, we decided to perform a graded drug provocation test. After infusion of 380 mg, he developed throat tightness, hand pruritus, dyspnea, wheezing, and his oxygen saturation went down to 84%. Symptoms were rapidly controlled with epinephrine. The patient was subsequently treated with vancomycin and as his clinical evolution was favorable, there was no indication to proceed with clindamycin desensitization. At follow-up 2 months later, skin prick test (SPT) and intradermal tests (IDT) were performed, and both were positive. Undiluted SPT with clindamycin (150 mg/ml)) was positive with an 8 mm wheal diameter and a negative saline control. IDTs were positive at dilutions of 10^−5^ and 10^−3^, with respective wheals of 10 mm and 12 mm with surrounding erythema compared to a negative IDT saline control. These dilutions were reported as non-irritating [7]. Because it was the first time that he received clindamycin antibiotherapy, we asked the parents to look for other sources of exposure to clindamycin. After verification, the mother reported the use of clindamycin gel for acne on one or two occasions in the previous year. Otherwise, this patient was known for previous asthma and peanut allergy during infancy, both of which completely resolved. He also presented a history of AD since childhood, for which he continues to apply daily moisturizing cream, tacrolimus 0.1% ointment and desoximetasone cream to maintain the control of his AD.

## Discussion

To our knowledge, this is the first report of intravenous clindamycin anaphylaxis with likely sensitization due to previous topical exposure to clindamycin gel. Although systemic treatment with clindamycin is generally considered as a second-line treatment or as an alternative in patients with suspected beta-lactam allergy, clindamycin is commonly used as a topical antibacterial cream or gel for acne vulgaris [1]. As both acne and AD are associated with skin inflammatory processes [8], the transcutaneous exposure to clindamycin through activated immune skin barrier may be a risk factor for sensitization, which could in this case explain patient’s IgE-mediated reaction to clindamycin upon reexposure.

Until now, only four cases of clindamycin anaphylaxis have been published since 1977 [7]. In those cases, previous exposure to clindamycin including topical forms was not specifically addressed by the authors. In our case, an IgE-mediated anaphylactic reaction to clindamycin was confirmed by both positive intradermal skin testing and drug challenge and the only risk factor found was previous transcutaneous exposure to topical clindamycin for treatment of acne.

The role and importance of the skin barrier in systemic sensitization is now better understood. Skin sensitization to different types of allergens such as plant components (latex [5]), topic antiseptics (chlorhexidine [6, 9]), cosmetic colorants (carmine red [10]) or foods (peanut [4]) is well described. In the last decades, many reports were published on severe anaphylactic reactions to natural rubber latex products in individuals with recurrent skin exposure to latex such as health-care workers or spina bifida patients [11]. The decrease in prevalence of latex allergy after the turn of the century correlates with reduced exposure to latex allergens after powdered latex gloves were banned in many medical centers [11].

Skin barrier is disrupted by inflammatory processes such as AD and acne, allowing entry of potential allergens and bacteria. In AD, activation of Langherans cells (LC) is known to stimulate the Th2-immune pathway, associated with the production of allergen-specific IgE, which explains why allergen exposure through disrupted skin barrier could allow sensitization and eventually cause an anaphylactic reaction upon allergen re-exposure [2, 3, 12]. In acne, the inflammatory process is closely associated with the skin microbiome, especially with the presence of *Propionibacterium acnes*. However, bacterial exposure generally stimulates a Th1-immune response, which could explain why transcutaneous drug sensitization is rare in acne. In this case, the patient had a history of atopy and mild AD. It has been documented that barrier and immune defects are still present in mild AD, such as increased transepidermal water loss, impaired lipids and increased T cell infiltrates [2]. Finally, systemic tacrolimus has also been reported as a risk factor for food-allergic occurrence in children post liver transplant [13]. It is not known whether topical tacrolimus may have contributed to drug sensitization in this case. In addition, the benefit of topical tacrolimus to reestablish the skin barrier integrity probably outgrows this hypothetical risk.

## Conclusion

This case highlights the potential sensitization to drug allergens via the skin when AD overlaps with acne. As both acne and AD affect a large portion of the teenage population and since clindamycin is a topical treatment frequently used, this potential adverse event may have to be increasingly taken into consideration by clinicians, especially when there is a previous history of AD. Further drug allergy studies will have to address the potential role of transcutaneous exposure in drug sensitization.

## Data Availability

The data used during the current report are available from the corresponding author on reasonable request.

## Abbreviations

AD: Atopic dermatitis
SPT: Skin prick test
IDR: Intradermal tests
LC: Langherans cells
